# Adropin and Irisin in Patients with Cardiac Cachexia

**DOI:** 10.5935/abc.20180109

**Published:** 2018-07

**Authors:** Ali Kemal Kalkan, Huseyin Altug Cakmak, Mehmet Erturk, Kübra Erol Kalkan, Fatih Uzun, Omer Tasbulak, Vesile Ornek Diker, Suleyman Aydin, Ahmet Celik

**Affiliations:** 1 Mehmet Akif Ersoy Thoracic and Cardiovascular Disease Education and Training Hospital, Department of Cardiology, Istanbul, Turkey; 2 Mustafakemalpasa State Hospital, Department of Cardiology, Bursa, Turkey; 3 Şişli Hamidiye Etfal Education And Research Hospital, Department of Internal Medicine, Istanbul, Turkey; 4 Mehmet Akif Ersoy Thoracic and Cardiovascular Disease Education and Training Hospital, Department of Biochemistry, Istanbul, Turkey; 5 Firat University, School of Medicine, Department of Clinical Biochemistry, Elazig, Turkey.; 6 Mersin University, School of Medicine, Department of Cardiology, Mersin, Turkey

**Keywords:** Cachexia / complications, Heart Failure / physiopathology, Hypertrophy, Left Ventricular, Ventricular Function, Left, Adropin, Peptides, Hormones

## Abstract

**Background:**

Cardiac cachexia is an important predictive factor of the reduction in survival of patients with heart failure with reduced ejection fraction.

**Objectives:**

The aims of the present study were to evaluate adropin and irisin levels in cachectic and non-cachectic subjects and the relationships between the levels of these proteins and clinical and laboratory parameters in patients with HFrEF.

**Methods:**

The clinical records of patients who were admitted to the cardiology outpatient clinic for heart failure with reduced ejection fraction were screened. Cachectic patients were identified and assigned to the study group (n = 44, mean age, 65.4 ± 11.2 y; 61.4% men). Heart failure with reduced ejection fraction patients without weight loss were enrolled as the control group (n = 42, mean age, 61.0 ± 16.5 y; 64.3% men). The serum adropin and irisin levels of all patients were measured. A p-value < 0.05 was considered significant.

**Results:**

Serum adropin and irisin levels were significantly higher in the cachexia group than in the controls (Adropin (ng/L); 286.1 (231.3-404.0) vs 213.7 (203.1-251.3); p < 0.001, Irisin (*µ*g/mL); 2.6 (2.2-4.4) vs 2.1 (1.8-2.4); p = 0.001). Serum adropin and irisin levels were positively correlated with brain natriuretic peptide (BNP) levels and New York Heart Association (NYHA) class and negatively correlated with body mass index (BMI) and serum albumin levels (all p values: < 0.001). In a multivariate analysis, adropin was the only independent predictor of cachexia in the heart failure with reduced ejection fraction patients (OR: 1.021; 95% CI: 1.004−1.038; p = 0.017).

**Conclusions:**

The results suggest that adropin and irisin may be novel markers of cardiac cachexia in heart failure with reduced ejection fraction patients. Adropin and irisin are related with the severity of heart failure.

## Introduction

Heart failure with reduced ejection fraction, which is a multifactorial and common disease, is considered a major public health problem worldwide.^1^ Cardiac cachexia, which is characterized by loss of muscle, with or without loss of fat mass, is a serious and life-threatening complication of heart failure with reduced ejection fraction. Moreover, studies have demonstrated that it was an important independent prognostic factor for cardiovascular mortality after adjustment for age, left ventricular ejection fraction and functional capacity to perform physical activities.^2-4^

Adropin is a novel membrane-bound protein, which contains 76 amino acids and is encoded by the energy homeostasis-associated gene.^5^ It is expressed predominantly in the liver, brain, coronary arteries, vascular endothelium and heart (all layers).^6^ A recent study reported that elevated plasma levels of adropin in heart failure with reduced ejection fraction patients were positively correlated with disease severity, as classified by the New York Heart Association (NYHA).^7^ Irisin is a thermogenic protein, which is expressed in adipose tissue, cardiac muscle, heart and other peripheral tissues. The main functions of irisin are energy expenditure by converting white adipose tissue to brown adipose tissue and regulation of carbohydrate metabolism, resulting in improved glucose homeostasis and insulin sensitivity and weight loss.^6-11^

Cardiac cachexia in heart failure with reduced ejection fraction is associated with impaired energy homeostasis due to anabolic and catabolic imbalance, and serum adropin and irisin levels play important roles in energy balance and metabolism. Based on the aforementioned, we hypothesized that both serum adropin and irisin levels would differ in cachectic heart failure with reduced ejection fraction patients and non-cachectic individuals.

The aims of the present study were: 1) to investigate serum adropin and irisin levels in cardiac cachectic and non-cachectic patients with heart failure with reduced ejection fraction, and 2) to investigate the relationship between adropin and irisin levels and clinical and laboratory parameters in patients with heart failure with reduced ejection fraction.

## Methods

### Patient selection and study protocol

To identify cachectic patients, the clinical records of patients admitted to the cardiology outpatient clinic of a training and research hospital either for the diagnosis or treatment of heart failure with reduced ejection fraction were screened. Subsequently, the patients were contacted by phone and asked to attend the clinic. Heart failure with reduced ejection fraction patients without weight loss were enrolled as a control group.

The inclusion criteria were a diagnosis of heart failure with reduced ejection fraction according to the ‘2012 ESC Guidelines for the Diagnosis and Treatment of Acute and Chronic Heart Failure’ and treatment for heart failure with reduced ejection fraction for at least 6 months before enrolment in the study.^12^ The following were exclusion criteria: acute decompensated heart failure, heart failure with preserved ejection fraction, hospitalization for acute coronary syndromes, primary valvular heart disease, chronic obstructive pulmonary disease, peripheral vascular disease, musculoskeletal disease, acute/chronic inflammatory or infectious diseases, connective tissue diseases, neoplastic diseases, congenital heart diseases, hepatic failure, acute or chronic end-stage kidney failure, recent trauma or major surgery and pregnancy.

Demographic, clinical and laboratory data and the medical therapies administered to each patient during their index hospitalization were recorded by a systematic review of the patient files. To determine left ventricle ejection fraction values, all individuals underwent a transthoracic echocardiographic examination (Vivid S5; General Electric, Wisconsin, USA), which was performed by an experienced operator. The left ventricle ejection fraction was determined using Simpson’s method of discs and two-dimensional echocardiography.

All patients were older than 18 years and able to provide written informed consent, which was a prerequisite for enrolment. The study complied with the Declaration of Helsinki, and the trial protocol was approved by the Local Ethical Committee.

### Laboratory measurements

Blood samples were drawn by venipuncture into tubes containing anticoagulant ethylenediaminetetraacetic acid (EDTA). The samples were collected after a 12-hour overnight fast from the antecubital vein, with the patient in a sitting position. The serum was obtained by centrifugation at 4000 rpm at 4°C for 20 min. The obtained sera were stored at -80°C until used in the analysis. All routine biochemical and hematological parameters were measured on the same day as the blood sampling. Biochemical parameters, including fasting blood glucose, creatinine, total cholesterol, high-density lipoprotein (HDL) cholesterol, low-density lipoprotein (LDL) cholesterol and triglycerides (TG), were measured using an Abbott Diagnostics C8000i (Abbott, Germany) auto-analyzer with commercial kits. The LDL cholesterol was assayed by applying Friedewald’s formula to samples with TG ≤ 400 mg/dL. Hematological parameters were obtained using a Coulter LH 780 Hematology Analyzer (Beckman Coulter Ireland, Inc., Mervue, Galway, Ireland). Serum brain-natriuretic peptide (BNP) levels (pg/ml) were measured using commercially available kits (Phoenix Pharmaceuticals, Inc., Burlingame, CA, USA).

Serum adropin levels were measured with a commercially available kit using an enzyme-linked immunosorbent assay (ELISA) method (Human adropin ELISA kit, catalogue n°. ck-e90267, Hangzhou Eastbiopharm Co., Blue Ocean International Times Mansion, China), with a low sensitivity limit of 2.49 ng/L. All samples were measured in duplicate in a single experiment. The intra- and inter-assay coefficients of variance of this kit were < 10% and < 12%, respectively. The detection range of adropin was 5-1000 ng/L. Serum irisin levels were detected with a commercially available kit, using the ELISA method (Human irisin ELISA kit, catalogue n°. CK-E90905, Hangzhou Eastbiopharm Co., Blue Ocean International Times Mansion, China). The sensitivity limit was 0.023 *µ*g/mL, and the intra- and inter-assay coefficients of variance were < 10% and < 12%, respectively. The detection range of irisin was 0.05-15 *µ*g/mL.

### Definitions

Cardiac cachexia can be defined as underlying disease and involuntary non-edematous weight loss ≥ 6% within the previous 6-12 month.^12,13^

Hypertension was diagnosed if systolic arterial pressure exceeded 140 mm Hg, diastolic arterial pressure exceeded 90 mmHg, or the patient was taking antihypertensive drugs. Hyperlipidemia was defined as fasting total serum cholesterol > 200 mg/dL, LDL cholesterol > 130 mg/dL, serum TG > 180 mg/dL or the use of lipid-lowering drugs. Diabetes mellitus was defined as a previous history of the disease, the use of insulin or oral antidiabetic drugs, or a fasting venous blood glucose level ≥ 126 mg/dL on two occasions in previously untreated patients.^14^ Anthropometric measurements were used to determine body mass index (BMI), triceps skinfold thickness (TST) and arm circumference (AC). The TST was measured using a Holtain skinfold caliper. The arm muscle area (AMA) was calculated by the formula (AC-TST × π) 2/4 × π and considered an indicator of body muscle mass.^15^ The heights and weights of the study participants were measured, and the BMI was calculated as body weight in kilograms divided by the square of the height in meters (kg/m^2^).

### Statistical Analysis

Descriptive analyses are presented using means and standard deviations or the median and the interquartile range (IQR, range from the 25th to 75th percentile). The standard effect size of the current trial was determined 0.62 with power of 80% and error of 5% according to the equation reported by Pardo et al.^16^ The sample size was established at a minimum of 41 volunteers per group to detect differences in irisin between cachectic and control patients.

The categorical variables are expressed as numbers and percentages. Visual (histograms and probability plots) and analytical methods (Kolmogorov-Smirnov) were used to determine whether the variables were normally distributed. The independent samples T-Test was used for the comparison of normally distributed continuous numerical variables, the Mann-Whitney *U*-test was used for non-normally distributed numerical variables, and the χ^2^-test was used for comparing categorical variables between the two groups. Receiver operating characteristic curves were plotted for BNP, adropin and irisin. When a significant cut-off value was observed, the sensitivity, specificity, positive and negative predictive values were recorded. Spearman’s correlation analysis was performed to determine the association of adropin and irisin levels with the examined variables. Multiple logistic regression analyses were performed to identify the independent risk factors associated with cachexia. Variables found to be statistically significant in the univariate analyses were entered into a multivariate logistic regression analysis. An overall 5% type-I error level was used to infer statistical significance, and a *p*-value less than 0.05 was considered significant. Statistical analyses were performed using the Statistical Package for Social Sciences (IBM SPSS 17 Statistics for Windows, Version 20.0. Armonk, NY, USA).

## Results

**Figure 1:**
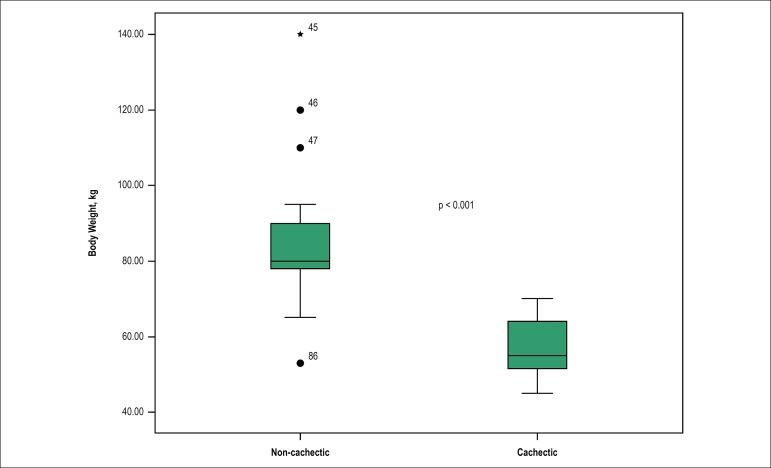
Body weight difference between cachectic and non-cachectic groups.

The present study included 86 heart failure with reduced ejection fraction patients: 44 with cardiac cachexia (mean age, 65.4 ± 11.2 y; 61.4% men) and 42 with a normal body weight (mean age, 61 ± 16.5 y; 64.3% men). The weight difference between two groups is shown in Figure 1. The baseline demographic and clinical characteristics of the study groups are summarized in Table 1. As expected, BMI, TST and AMA were significantly lower in the cardiac cachexia group than the non-cachectic group. The NYHA class of the two groups was also significantly different, with more patients in the cardiac cachexia group classified as NYHA class III and IV, and more in the non-cachectic group classified as NYHA class I and II.

**Table 1:** Baseline demographic, clinical and laboratory characteristics of the study groups

	CHF without cachexia (n = 42)	CHF with cachexia (n = 44)	p value
Age, (years), mean (SD)	61.0 (16.47)	65.4 (11.18)	0.179
Male Gender, n (%)	27 (64.3)	27 (61.4)	0.779
NYHA, class I-II, n (%)	30 (60)	20 (40)	0.015
class III-IV, n (%)	12 (33.3)	24 (66.7)	
Ischemic Etiology, n (%)	28 (66.7)	26 (59.1)	0.468
**LVEF, n (%)**			
Anthropometric parameters	31.7 (7.89)	31.4 (6.71)	0.882
BMI (kg/m^2^), mean (SD)	29.2 (4.25)	19.9 (1.12)	< 0.001
TST (mm), mean (SD)	17.9 (3)	13.4 (2.45)	< 0.001
AMA (cm^2^), mean (SD)	35.9 (8.7)	24.4 (4.03)	< 0001
**Comorbidities**			
Hypertension, n (%)	27 (64.3)	26 (59)	0.620
Diabetes mellitus, n (%)	18 (42.9)	26 (59.1)	0.290
Chronic renal failure, n (%)	14 (33.3)	15 (34.1)	0.941
Chronic obstructive lung disease, n (%)	8 (19)	8 (18.2)	0.918
**Laboratory parameters**			
Glucose (mg/dL), mean (SD)	155.3 (78.5)	150.3 (48.3)	0.685
Creatinine (mg/dL), mean (SD)	1.15 (0.63)	1.19 (0.8)	0.997
Hemoglobine (%), mean (SD)	11.9 (1.38)	11.3 (1.34)	0.049
WBC (mg/L), mean (SD)	8.35 (4.2)	8.45 (3.98)	0.742
Adropin (ng/L) median (IQR)	213.7 (203.1-251.3)	286.1 (231.3-404.0)	< 0.001*
Irisin ( µg/mL), median (IQR)	2.1 (1.8-2.4)	2.6 (2.2-4.4)	0.001*
BNP (pg/mL), median (IQR)	698.0 (340.0-1517.0)	1408.5 (725.0-4041.0)	0.001*
Albumin (mg/dL), mean (SD)	3.3 (0.46)	3.12 (0.36)	0.041
Sodium (mEq/L), mean (SD)	138.7 (10.1)	135.7 (9.7)	0.136
Total cholesterol (mg/dL), mean (SD)	164.5 (44.1)	153.2 (44.4)	0.240
LDL-cholesterol (mg/dL), mean (SD)	108.4 (40.5)	101.1 (32.7)	0.366
HDL-cholesterol (mg/dL), mean (SD)	36.2 (10.4)	31 (9.1)	0.015
**Triglyceride (mg/dL), mean (SD)**			
Drug therapy	134.2 (50)	122.3 (56)	0.302
Furosemide, n (%)	35 (83.3)	40 (90.9)	0.293
ACE-i/ARB, n (%)	20 (47.6) / 11 (26.2)	28 (63.6) / 11 (25.2)	0.136
Spironolactone, n (%)	26 (61.9)	30 (68.2)	0.542
Statin, n (%)	16 (38.1)	18 (40.9)	0.790
Beta-blocker, n (%)	33 (78.6)	39 (88.6)	0.206
Ivabradine, n (%)	15 (25)	11 (25)	0.289
CRT, n (%)	9 (21.4)	6 (13.6)	0.341

n: number; SD: standard deviation; IQR: interquartile range; NYHA: New York Heart Association; LVEF: left ventricular ejection fraction; BMI: body mass index; TST; triceps skinfold thickness; AMA: arm muscle area; WBC: white blood cell; BNP: brain natriuretic peptide; LDL: low-density lipoprotein; HDL: high-density lipoprotein; ACE-i: angiotensin-converting-enzyme inhibitor; ARB: angiotensin receptor blocker; CRT: cardiac resynchronization therapy.

* mann-whitney u-test.

The baseline laboratory characteristics of the two groups are presented in Table 1. Hemoglobin, albumin and HDL cholesterol levels were significantly higher in the non-cachectic individuals compared to the cachectic patients. Furthermore, the serum BNP, adropin and irisin levels were significantly higher in the cachectic group than in the non-cachectic group [adropin (ng/L): 286.1 (231.3-404.0) vs 213.7 (203.1-251.3), p < 0.001; irisin (*µ*g/mL): 2.6 (2.2-4.4) vs 2.1 (1.8-2.4), p = 0.001; BNP (pg/mL): 698.0 (340.0-1517.0) vs 1408.5 (725.0-4041.0), p = 0.001]. Analysis of the association between adropin and irisin levels and the clinical and laboratory parameters of the patients (Table 2) revealed that NYHA class and BNP levels were significantly positively correlated with both adropin and irisin levels. However, BMI, AMA, TST and serum albumin, which were significant indirect clinical and laboratory indicators of cardiac cachexia, were significantly inversely correlated with adropin and irisin levels. In addition, there was a direct correlation between adropin and irisin levels and heart failure with reduced ejection fraction. Creatinine levels were also positively correlated with irisin levels.

**Table 2:** The correlations of adropin and irisin with clinical and laboratory parameters of patients

		Age	BMI	AMA	TST	Albumin	BNP	NHYA	Irisin	LVEF	Creatinine
Adropin	r	0.077	-0.463	-0.386	-0.415	-0.250	0.676	0.762	0.669	-0.042	0.177
	p	0.480	< 0.001	< 0.001	< 0.001	0.02	< 0.001	< 0.001	< 0.001	0.704	0.104
Irisin	r	0.044	-0.384	-0.279	-0.374	-0.323	0.403	0.523		0.123	0.232
	p	0.687	< 0.001	< 0.001	< 0.001	0.002	< 0.001	< 0.001		0.259	0.031

BMI: body mass index; AMA: arm muscle area; TST: triceps skinfold thickness; BNP: brain natriuretic peptide; NYHA: New York Heart Association; LVEF: left ventricular ejection fraction.

**Figure 2:**
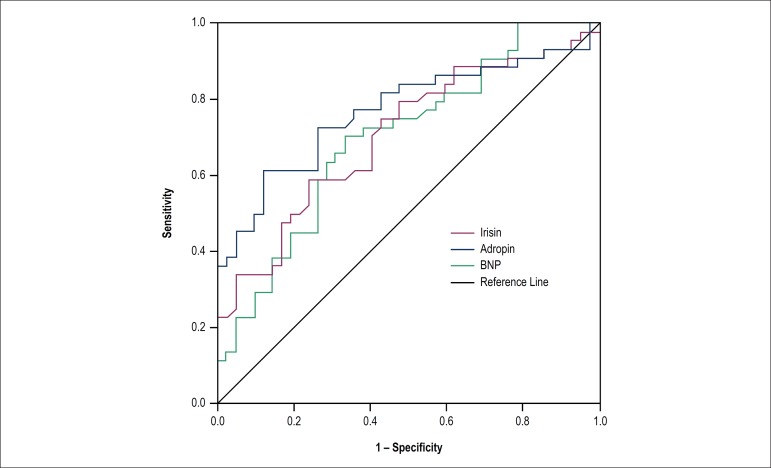
Receiver-operating characteristic curve for discriminative value of serum adropin, irisin and BNP levels in systolic heart failure with reduced ejection fraction patients with or without cachexia.

To investigate the discriminative value of serum BNP, adropin and irisin in cachectic and non-cachectic heart failure with reduced ejection fraction patients, a receiver operator characteristic curve was generated for sensitivity and specificity, using the respective areas under the curve (AUC) (Figure 2 and Table 3). The results indicated that adropin levels greater than 229.4 pg/mL had sensitivity of 77.3% and specificity of 64.3% for cardiac cachexia in heart failure with reduced ejection fraction patients [AUC: 0.770; 95% confidence interval (CI): 0.668-0.872; p < 0.001]. Moreover, the sensitivity of irisin levels of more than 2.2 pg/mL was 75.0%, whereas the specificity was 52.4% for cachexia (AUC: 0.705; 95% CI: 0.596-0.815; p < 0.001).

**Table 3:** Receiver-operating characteristic curve analysis of adropin, irisin and brain natriuretic peptide (BNP) for predicting cachexia

Variable	AUC	SE	CI (95%)	P value	Sensitivity	Specificity	PPV	NPV
Adropin	0.770	0.052	0.668-0.872	0.0001	%77.3	%64.3	%69.4	%73.0
Irisin	0.705	0.056	0.596-0.815	0.001	75.0	%52.4	%62.3	%66.7
BNP	0.700	0.056	0.590-0.811	0.001	%72.7	%61.9	66.7	%68.4

AUC: area under the curve; SE: standard error; PPV: positive predictive value; NPV: negative predictive value.

Variables found to be statistically significant in the univariate analyses were entered into a multivariate logistic regression analysis. In the multivariate analysis, adropin [odds ratio (OR) 1.021, 95% CI: 1.004-1.038; p = 0.017] was the only independent predictor of the presence of cachexia in patients with heart failure with reduced ejection fraction (Table 4).

**Table 4:** Logistic regression analyses to identify the independent risk factors associated with cardiac cachexia

	Univariate			Multivariate		
	p	OR	(95% CI)	p	OR	(95% CI)
Albumin	0.044	0.331	0.113-0.972	0.387	0.571	0.161-2.029
BNP	0.013	1.000	1.000-1.001	0.770	1.000	1.000-1.000
Age	0.151	1.023	0.992-1.056			
Gender	0.779	1.133	0.472-2.720			
Irisin	0.025	1.865	1.081-3.218	0.776	0.880	0.378-2.047
Adropin	0.002	1.016	1.006-1.026	0.017	1.021	1.004-1.038
Creatinine	0.760	1.098	0.604-1.994			
Glucose	0.720	0.999	0.992-1.005			
LVEF	0.880	0.996	0.939-1.056			
Total cholesterol	0.239	0.994	0.984-1.004			
Triglyceride	0.302	0.996	0.987-1.004			
LDL	0.363	0.995	0.983-1.006			
HDL	0.022	0.941	0.893-0.991	0.102	0.950	0.893-1.010
NYHA III - IV	0.016	3.000	1.226-7.339	0.463	0.550	0.111-2.717

BNP: Brain Natriuretic Peptide; LVEF: Left Ventricular Ejection Fraction; LDL: Low-Density Lipoprotein; HDL: High-Density Lipoprotein; NYHA: New York Heart Association.

## Discussion

The main findings of the study were as follows: 1) serum adropin and irisin levels were significantly higher in the cachexia group than in the non-cachectic subjects; 2) NYHA class and BNP levels, which are validated indicators of heart failure with reduced ejection fraction severity, were significantly positively associated with both adropin and irisin levels; 3) there was a direct relation between adropin and irisin levels; 4) sensitivity of adropin and irisin were higher than their specificity for predicting cardiac cachexia. Both adropin and irisin sensitivity higher than BNPs sensitivity; and 5) adropin was the only independent predictor of the presence of cachexia in patients with heart failure with reduced ejection fraction.

The annual incidence of cardiac cachexia in patients with NYHA class III-IV was reported to be 10%, and the prevalence was reported to be 12-15% among those with NYHA class II-IV.^13^ Several factors, including impaired food intake and absorption, immunological and neurohormonal activation, endothelial dysfunction, increased insulin resistance, triggered pro-inflammatory cytokine production and anabolic and catabolic imbalance, play a pivotal role in the complex process of cardiac cachexia.^13,17^ This complex is associated with poor short- and long-term prognoses, unfavorable response to drug treatment and poor quality of life.^18^ Previous studies reported elevated levels of some hormones and peptides, such as adiponectin, ghrelin, leptin and melanocortin, in cachectic heart failure with reduced ejection fraction patients.^17,19-20^ However, there are no studies on the levels of adropin and irisin in this patient population in the literature. In the present study, the levels were significantly elevated in the cardiac cachexia group with heart failure with reduced ejection fraction compared to the non-cachectic group.

Sente et al.^21^ have reported that cardiac and skeletal muscle energy deficiency played a major role in the pathophysiology of heart failure, which results in a hyperadrenergic state. Plasma free fatty acids increase under a hyperadrenergic state and inhibit glycolysis and glucose uptake by heart and skeletal muscle, with subsequent increases in plasma glucose. Multifactorial pancreatic damage, together with hyperglycemia, causes both systemic and myocardial insulin resistance.^22^ The concept of metabolic failure in heart failure with reduced ejection fraction includes both catabolic over-reactivity (lipolysis) and anabolic deficiency, with catabolic over-reactivity activating glycolytic and lipolytic pathways and anabolic deficiency inducing loss of skeletal muscle mass and function.^18^

Adropin is a recently identified protein, which has been implicated in the maintenance of energy homeostasis.^5^ A study of adropin-deficient mice suggested that this peptide hormone was required for maintaining insulin sensitivity and protecting against impaired glucose tolerance.^23^ Thus, we hypothesized that adropin might increase as a consequence of insulin resistance in heart failure with reduced ejection fraction patients.

Kumar et al.^5^ have reported that overexpression or systemic administration of adropin in diet-induced obese mice resulted in a marked improvement in insulin sensitivity and weight loss. Thus, weight loss in cachectic heart failure with reduced ejection fraction patients could contribute to the elevation of plasma adropin levels. The findings of the present study pointed to a metabolic association of increased serum adropin with muscle wasting and lipolysis in cachectic heart failure with reduced ejection fraction patients.

In addition to important metabolic effects of adropin, Lovren et al. have reported a potential endothelial protective role for this protein that was likely mediated by upregulation of endothelial nitric oxide synthase (eNOS) expression. They suggested that adropin might help protect against vascular diseases by markedly elevating eNOS expression of coronary artery endothelial cells.^24^ Topuz et al.^9^ have reported reduced adropin levels in type 2 diabetic patients with endothelial dysfunction. Wu et al.^8^ have demonstrated an inverse and independent association between adropin levels and the severity of coronary artery atherosclerosis in diabetic patients. Zhang et al.^25^ have presented similar results for patients with stable coronary artery disease. In another study, they have reported an important association between decreased adropin levels, high SYNTHAX scores and the severity of stable coronary artery disease.^26^ Yu et al.^27^ have examined the role of adropin in acute myocardial infarction (MI) and have shown that serum adropin levels were reduced in cases of acute MI.

By elevating eNOS, adropin may have the potential to improve endothelial dysfunction, which has been widely reported in patients with heart failure with reduced ejection fraction, and decelerate left ventricular dysfunction in heart failure with reduced ejection fraction.^28^ Lian et al.^7^ have reported that an elevated level of adropin in heart failure with reduced ejection fraction was correlated with the severity of heart failure with reduced ejection fraction according to the NYHA class and BNP levels. The present study revealed similar findings and relations in cachectic patients with heart failure with reduced ejection fraction. Unlike the study by Lian et al., in which adropin levels and BMI were directly correlated with each other, there was an inverse relationship between adropin levels and BMI in cardiac cachexia in the present study, as expected.

Although irisin is predominantly expressed in muscle and is directly associated with muscle mass, it can be expressed in different tissues. Brown adipose tissue is known to dissipate energy in the form of heat via activation of uncoupling protein 1. This process increases energy expenditure, reduces body weight and improves metabolic parameters, such as insulin sensitivity. In white tissue, irisin stimulates BAT-like phenotype changes via a process known as browning. Based on the aforementioned properties, irisin has been proposed as a possible novel treatment for diabetes and obesity.^29^ Although some studies have reported positive correlations between irisin and BMI, others have reported contradictory results.^6,29^ The present study revealed an inverse correlation between irisin and BMI. In addition, AMA, TST and serum albumin levels were inversely related with irisin. In patients with heart failure with reduced ejection fraction, muscle, fat and bone loss were reported to be associated with worse outcomes.^30^ Moreover, a recent study reported a gradual decrease in irisin levels in patients with acute MI, suggesting that irisin may be a new diagnostic marker in this setting.^31^ In a recently published study, Shen et al.32 have reported that serum irisin level was significantly higher in deceased acute heart failure (AHF) patients compared to that in survived AHF and predicted 1-year all-cause mortality in AHF patients. In that study irisin and NT-pro-BNP were determined by ROC curve analysis. NT-pro-BNP (AUC: 0.670) had only moderate prognostic values for AHF mortality risk compared to serum irisin level (AUC: 0.753).^32^ The findings of that study are similar to ours. This increase may be the result of adipose tissue metabolism and insulin resistance. Studies are needed to determine whether irisin levels are the result of a reduced peripheral muscle mass in cachectic heart failure with reduced ejection fraction patients. In addition, in our study, adropin was found to be more predictive than irisin and BNP.

In our study, only adropin was found to be an independent predictor of cachexia in patients with heart failure. Although irisin predicted cardiac cachexia in univariate analysis, it did not predict in multivariate analysis. Irisin was found to be a predictive biomarker for 1-year all-cause mortality in the study by Shen et al.^32^ This difference may be due to the fact that the adropin molecule was not used in multivariate analysis at this work. Further studies are highly needed to examine this relationship.

Similar to adropin, irisin was significantly positively correlated with BNP levels and NYHA class. Natriuretic peptides, such as BNPs, in addition to diuretic peptides and vasodilators, trigger lipolysis in the human body and play a role in fat metabolism.^7^ Hence, we hypothesized that lipolysis by BNPs might be associated with adropin and irisin synthesis in cachectic heart failure with reduced ejection fraction patients. A further study will be necessary to elucidate the precise mechanism of adropin and irisin release in patients with cardiac cachexia.

### Study Limitations

The present study had some limitations. Firstly, the study population was relatively small. However, the results pointed to an important relationship between adropin and irisin levels and cardiac cachexia in patients with heart failure with reduced ejection fraction. Secondly, a lack of follow-up data on future major adverse cardiovascular events, including mortality or hospitalization for heart failure with reduced ejection fraction, meant that the prognostic value of the levels of both proteins could not be evaluated.

## Conclusions

The present study showed that serum adropin and irisin levels were significantly increased in the cachectic heart failure with reduced ejection fraction group and that these were significantly associated with previously validated markers of heart failure with reduced ejection fraction severity, such as the BNP level and NYHA class. The results suggest that adropin and irisin may be novel markers of cardiac cachexia in heart failure with reduced ejection fraction patients. Adropin and irisin are related with the severity of heart failure.
